# Exploring ChatGPT’s potential in the clinical stream of neurorehabilitation

**DOI:** 10.3389/frai.2024.1407905

**Published:** 2024-06-06

**Authors:** Maria Grazia Maggio, Gennaro Tartarisco, Davide Cardile, Mirjam Bonanno, Roberta Bruschetta, Loris Pignolo, Giovanni Pioggia, Rocco Salvatore Calabrò, Antonio Cerasa

**Affiliations:** ^1^IRCCS Centro Neurolesi Bonino-Pulejo, Messina, Italy; ^2^Institute for Biomedical Research and Innovation (IRIB), National Research Council of Italy (CNR), Messina, Italy; ^3^S’Anna Institute, Crotone, Italy; ^4^Pharmacotechnology Documentation and Transfer Unit, Preclinical and Translational Pharmacology, Department of Pharmacy, Health and Nutritional Sciences, University of Calabria, Rende, Italy

**Keywords:** ChatGPT, generative AI, acquired brain injury, neurorehabilitation, artificial intelligence

## Abstract

In several medical fields, generative AI tools such as ChatGPT have achieved optimal performance in identifying correct diagnoses only by evaluating narrative clinical descriptions of cases. The most active fields of application include oncology and COVID-19-related symptoms, with preliminary relevant results also in psychiatric and neurological domains. This scoping review aims to introduce the arrival of ChatGPT applications in neurorehabilitation practice, where such AI-driven solutions have the potential to revolutionize patient care and assistance. First, a comprehensive overview of ChatGPT, including its design, and potential applications in medicine is provided. Second, the remarkable natural language processing skills and limitations of these models are examined with a focus on their use in neurorehabilitation. In this context, we present two case scenarios to evaluate ChatGPT ability to resolve higher-order clinical reasoning. Overall, we provide support to the first evidence that generative AI can meaningfully integrate as a *facilitator* into neurorehabilitation practice, aiding physicians in defining increasingly efficacious diagnostic and personalized prognostic plans.

## 1 Introduction

Nearly two decades ago, medical science experienced a significant upheaval with the emergence of Artificial Intelligence (AI). The development of AI algorithms aimed at assisting doctors in diagnosing patients, selecting treatments, and predicting outcomes stemmed from the increasing necessity to tackle complex clinical challenges and effectively analyze and utilize vast amounts of accumulated knowledge. AI systems have been developed with the intention of simplifying the daily tasks of healthcare personnel, particularly in jobs involving the manipulation of data and knowledge (Ramesh et al., 2004).

After several years and thousands of empirical studies later, we can now affirm that AI has not merely transformed medical science; rather, it has significantly enhanced it. Several tools, devices, and methods have been developed, created, and commercialized exploiting AI-related algorithms with the aim of improving and standardizing clinical diagnosis and prognosis (Beam et al., 2023). The application of medical AI systems in routine clinical care today offers a significant although mainly unrealized potential. In fact, the medical AI community is actively addressing the numerous ethical, technological, and human-centered challenges necessary for safe and efficient translation.

Over the past two years, a new AI-related solution is poising to reshape medicine, the Generative Pre-Trained Transformer (GPT), an AI language model created by OpenAI. Recent demonstrations have shown that these models have exceptional natural language processing (NLP) skills, enabling them to excel in sophisticated case analysis and free-text clinical reasoning, as well as accurately answering multiple-choice medical questions (Cerasa and Crowe, 2024). Indeed, ChatGPT can comprehend and produce text that closely resembles that of a human being. Due to its unique capabilities, ChatGPT is well-suited for various applications, such as customer service, content creation, and even rehabilitation therapy (Clusmann et al., 2023; Kanjee et al., 2023).

In the realm of acquired brain injury (ABI), which affects millions of people globally, creating a rehabilitation plan poses an extremely intricate challenge. Indeed, many demographic, biological, neurophysiological, neuropsychological, psychological/psychiatric, and clinical factors should be considered in order to determine the best-personalized rehabilitation plan. Furthermore, individual recovery patterns vary widely, and the outcomes of ABI patients are highly variable, making it challenging to determine an optimal clinical course trajectory (Panunzi et al., 2023). These plans are intended to boost physical function, lessen impairment, improve older patients’ quality of life who are fragile or have multiple medical conditions, and encourage healthy living. To reach this target, these therapies necessitate the knowledge of skilled experts who interact directly and continuously with patients and their caregivers. Currently, there is a gap between the demand and supply for rehabilitation services, resulting in lengthy wait times and limited access to therapy for many patients. Furthermore, this shortage is exacerbated by the global scarcity of specialists in medical rehabilitation units. Indeed, as claimed by Ishikawa (2020), there is an urgent need for younger physicians to enter the field to address the significantly increasing demands for rehabilitation, driven by the aging population.

In the era of personalized medicine and remote assistance, the arrival of ChatGPT in neurorehabilitation practice could help to overcome these issues (Eriksen et al., 2024). Healthcare providers could supplement conventional therapy approaches by integrating ChatGPT into rehabilitation therapy programs, offering patients an AI-driven therapeutic strategy. ChatGPT can provide patients with interactive, individualized assistance, keeping them motivated and engaged in the healing process (Chakraborty et al., 2023). For instance, ChatGPT can monitor the progress of recovery from physical injuries, recommend exercises, and provide feedback to patients. Through conversation, ChatGPT can also help patients improve their language abilities, which is especially beneficial for individuals recovering from a stroke or traumatic brain damage. Additionally, ChatGPT is a user-friendly tool that patients can use, as it is accessible and convenient to use on computers or cell phones (Chakraborty et al., 2023; Eriksen et al., 2024).

This scoping review outlines the architecture underlying the functioning of ChatGPT and its application in clinical medicine. Moreover, we investigate its potential role in neurorehabilitation practice, considering the available literature.

### 1.1 ChatGPT: architecture and functioning

ChatGPT, developed by OpenAI, is a chatbot application based on the large language model GPT-3. It is designed to perform a variety of tasks such as text completion, translation, and question-answering (Nova, n.d.). The architecture of ChatGPT is a deep neural network composed of many layers of neurons, which are processing units. Each neuron receives information from the previous layer and then produces an output that is sent to the subsequent layer (Chakraborty et al., 2023).

**Figure 1:**
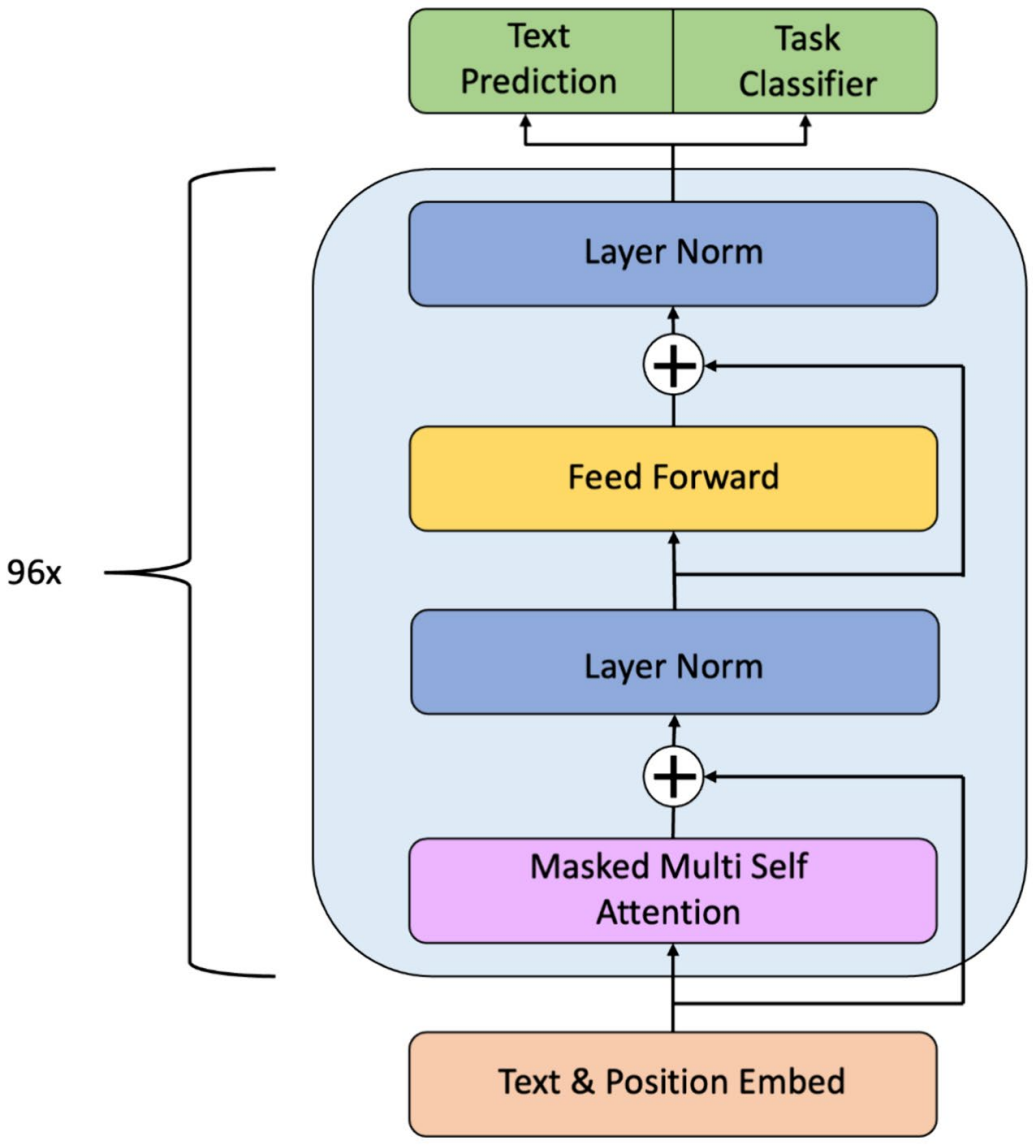
Overall architecture of GPT-3. The hierarchical structure is made up of different layers of transformer decoding, each of which is responsible for a different phase of text processing. The embedding layer converts the input tokens into numerical vectors, the normalization layer stabilizes the training process, the feed-forward layer captures complex patterns in the input data, and the masked multi-head self-attention layer captures the relationship between different tokens in the input sequence.

The transformer architecture used by ChatGPT, first described in the Vaswani et al. paper (Nova, n.d.), consists of multiple layers of self-attention and feed-forward neural networks. Each layer processes input from the previous layer, enabling the model to capture increasingly complex patterns and dependencies in the input data (Lee et al., 2023). The encoder and the decoder are the two key parts of the transformer architecture. The input text is processed by the encoder, which creates a set of encoded representations; the input of the decoder, which creates the output text, is these encoded representations. Specifically, the GPT-3 architecture is based on Decoder-only transformers (Figure 1). ChatGPT architecture is similar to GPT-3, although smaller in training size. To tailor it for use as a chatbot, it underwent further fine-tuning through a transfer learning approach.

The development of ChatGPT involved a two-phase process: unsupervised pre-training followed by supervised fine-tuning (Lee et al., 2023). During the pre-training phase, the model was exposed to a vast corpus of text including licensed data, human-generated content, and publicly available information from diverse domains such as books, websites, and Wikipedia (Bandi et al., 2023; Ray, 2023), using language modeling and masked language modeling techniques (Lee et al., 2023). Following pre-training, the model underwent supervised fine-tuning, during which OpenAI trainers assumed dual roles as human users and AI bots. Through dialogues designed to emulate human communication, the model’s dataset was enriched to enhance its conversational capabilities (Bandi et al., 2023; Ray, 2023). Subsequent improvements of ChatGPT included the development of a reward model for reinforcement learning from human feedback (RLHF) (Bandi et al., 2023).

### 1.2 The ChatGPT in medicine

Over the past two years, some relevant viewpoints have emerged about the impact of ChatGPT’s introduction into medical care. An editorial published in *Nature Medicine* (Editorial Nature, 2023), emphasizes that AI-based NLP has the potential to enhance patient care, quality of life, and healthcare delivery, as well as medical education. In particular, ChatGPT could readily evolve into a mobile mental health app for physicians and postgraduate students (Graber-Stiehl, 2023).

Initial empirical evidence about the potential of ChatGPT in medical care would seem to confirm this early impression. Indeed, in a recent study published in the *New England Journal of Medicine*, Eriksen et al. (2024) evaluated how well the GPT-4 performed in diagnosing difficult medical case problems and compared its success rate with that of medical journal readers. GPT-4 outperformed 99.98% of simulated human readers created from online answers, accurately diagnosing 57% of cases. This preliminary evidence was also confirmed by other authors using also different ChatGPT versions. Kung et al. (2023) demonstrated satisfactory accuracy (60%) on standardized medical examinations using ChatGPT 3.0, without the specific guidance of a human teacher. They employed 376 publicly available test questions released, retrieved from the United States Medical Licensing exam website. Fonseca et al. (2024) used ChatGPT 3.5 in the clinical diagnosis of neurological disorders, comparing its performance with that of 1,500 neurologists. These authors assessed the accuracy of diagnosis starting from a pool of clinical cases extracted from the American Academy of Neurology’s Question of the Day application. The AI chatbot showed a mean success rate of 71.3% in answering 188 questions correctly. Moreover, the AI chatbot provided the correct diagnosis in 85.0% of clinical questions, and in 96.1% of cases, it provided a satisfactory explanation for its answers. Finally, Kanjee et al. (2023) examined the performance of GPT in clinical settings requiring sophisticated diagnostic reasoning. The authors assessed the accuracy of GPT-4 in several clinically diagnostic cases by analyzing *New England Journal of Medicine* clinicopathological case conferences. They demonstrated that the generative AI model correctly identified the diagnosis in its differential in 64% of challenging cases and as its top diagnosis in 39%.

## 2 The ChatGPT in (neuro-)rehabilitation: a mini-review

As previously noted, there has been a growing interest in recent years in the application of ChatGPT in psychiatric (Cheng et al., 2023a) and neurological domains (Cerasa and Crowe, 2024), with promising results documented in the literature. ChatGPT has shown potential in aiding diagnosis, treatment planning, and even providing therapy in these fields. Nevertheless, despite these advancements, there is a notable gap in the literature concerning the specific application of ChatGPT within the field of neurorehabilitation. While ChatGPT’s capabilities in psychiatric and neurological contexts are increasingly recognized, its application in neurorehabilitation, which involves a distinct set of challenges and objectives, has not been thoroughly explored. In the psychiatric and neurological fields, the primary focus is on understanding and treating disorders related to mental health and the central nervous system, respectively (Cheng et al., 2023a; Clusmann et al., 2023; Fonseca et al., 2024). Psychiatric disorders encompass conditions such as depression, schizophrenia, and bipolar disorder, which primarily affect cognition, mood, and behavior (Cheng et al., 2023a). In contrast, neurological disorders such as Parkinson’s disease, stroke, and epilepsy, affect the structure or function of the brain and peripheral nervous system, resulting in motor, sensory, or cognitive impairments. Neurorehabilitation, however, entails the recovery and restoration of function in individuals with neurological disorders. Traditional psychiatric or neurological interventions focus on symptom management or pharmacological treatment. In contrast, neurorehabilitation emphasizes restoring lost function, improving independence, and enhancing quality of life through various therapeutic modalities. These modalities may include physical therapy, occupational therapy, speech therapy, cognitive rehabilitation, brain stimulation, and assistive technologies tailored to address specific deficits caused by neurological damage or disease. Thus, while there may be overlaps between psychiatric, neurological, and neuro-rehabilitation domains, the latter is distinct in its focus on restoring and optimizing function following neurological insult or injury.

To comprehend the current state of ChatGPT utilization in neurorehabilitation, we conducted a comprehensive search, according to the PRISMA-ScR criteria (Tricco et al., 2018). The PCC (Population/Problem, Concept, Context) model (Pollock et al., 2023) was used to define the research question. In terms of population/problem, our focus is on individuals undergoing neurorehabilitation. The central concept, ChatGPT, encompasses its applications in: (a) providing medical diagnosis and decision support for neurorehabilitation treatment; (b) its influence on patient engagement and motivation; (c) its personalization for individual patient needs; and (d) its integration with existing neurorehabilitation strategies and technologies. Regarding context, our review encompasses diverse settings in which neurorehabilitation occurs. These settings include clinical environments like hospitals and rehabilitation centers, home neurorehabilitation, virtual or telerehabilitation facilities, and various neurological conditions such as stroke or traumatic brain injury. Our objective is to comprehend the current understanding of ChatGPT’s role in neurorehabilitation.

A review was conducted for all peer-reviewed articles published from January 2020 through April 2024, using the following terms [(ChatGPT) AND (Rehabilitation)]. A review of currently published studies was performed in the following databases: PubMed, Scopus, and IEEE Xplore. All search results were imported into an online database (RYYAN) and screened by two blinding reviewers (MM and MB) to minimize the risk of bias (e.g., publication bias, delay bias, language bias). After screening based on titles and abstracts, the blind was opened and in case of disagreement, the other two reviewers were included in the decision process (AC and RCS). All articles were initially reviewed based on titles and abstracts by two investigators (MM and MB). The list of articles was then refined for relevance, revised, and summarized, with the key topics identified from the summary based on the inclusion/exclusion criteria.

The inclusion criteria were: (i) adult patients with acquired brain injury (ABI) and neurodegenerative disorders; (ii) studies described or investigated the use of ChatGPT in neurorehabilitation; (iii) the English language; and (v) published in a peer-reviewed journal. We have excluded articles describing theoretical models, methodological approaches, algorithms, and basic technical descriptions. Additionally, we excluded: (i) animal studies; (ii) conference proceedings or reviews; and (iii) studies involving children.

Following a preliminary search, 202 articles were found. After removing duplicates, 93 articles remained. Furthermore, 88 articles were excluded because they did not meet the inclusion criteria (dealing with education, rheumatic diseases, optology, or neurosurgery). The remaining 21 articles were submitted to the externally supervised reviewer (RCS and AC) because there was 95% agreement between the two reviewers and some of the included articles were of questionable inclusion for both (MM and MB).

After an in-depth analysis, only 12 articles were relevant to the research objectives and met the criteria.

In the first study, Sarraju et al. (2023) explored the efficacy of ChatGPT in responding to basic inquiries regarding cardiovascular disease prevention, including those related to cerebral ischemia and heart attacks. The results suggest that the AI model generally provides satisfactory responses, indicating its potential to aid in patient education and communication between patients and healthcare providers to promote disease prevention. In a cross-sectional study conducted by Sinha et al. (2023) the authors highlighted ChatGPT’s 75% accuracy rate in resolving one hundred random questions, covering cardiovascular disease, as well as other areas of pathology and internal medicine. This aspect was further explored by Kung et al. (2023) who observed that ChatGPT exhibits competence in executing various intricate tasks related to the management of complex medical and clinical information. In their study, they assessed ChatGPT’s capabilities in addressing biomedical and clinical questions characterized by standardized complexity and difficulty. Finally, Lu et al. (2023) observed that ChatGPT, when applied to support intensive care unit medicine, serves a range of purposes, including knowledge augmentation, device management, clinical decision-making support, early warning systems, and the establishment of an intensive care unit (ICU) database. However, Gianola et al. (2024) found ChatGPT (GPT-3.5) had poor internal consistency and accuracy compared to the clinical practice guidelines for diagnosing and treating lumbosacral radicular pain. To this end, the authors suggested that ChatGPT should be used with caution in the clinical routine, since its recommendations may be misleading.

Furthermore, recent studies conducted by Roy et al. (2024) and Suri et al. (2024) highlight the promising potential of ChatGPT in facilitating communication and delivering personalized support to neurorehabilitation patients. In line with these studies, Cheng et al. (2023b), Brown et al. (2023), and Mittal and Dhar (2023) have defined tangible examples of ChatGPT utilization and its impact on patient outcomes. Specifically, Mittal and Dhar (2023) presented a case involving a hypertensive patient undergoing post-stroke neurorehabilitation. ChatGPT was able to provide a complete and tailored rehabilitation program like those typically administered by clinicians. Finally, some authors evaluated the benefits of ChatGPT, including enhancements in communication and treatment adherence throughout the recovery process (Roman et al., 2023; Chen et al., 2024; Rodriguez et al., 2024).

In conclusion, despite the limited studies ChatGPT demonstrates promising potential in supporting various aspects of healthcare, from addressing pivotal questions in cardiovascular disease prevention to excelling in the diagnosis of complex medical challenges, including patient education, communication enhancement between patients and medical professionals, and intensive care unit medicine applications. Collectively, this preliminary evidence stimulates new studies on the diverse applications of ChatGPT in advancing medical knowledge, decision-making, and overall healthcare management.

## 3 ChatGPT & physicians: clinical case scenarios

To assess ChatGPT’s performance in neurorehabilitation practice, we tested its extensive semantic medical knowledge and capacity for medical reasoning on two medical cases, already published by our group (Calabrò et al., 2017; Maggio et al., 2020) (Boxes 1, 2). We utilized these case scenarios to prompt responses from the ChatGPT 3.5 version to questions posed by senior healthcare physicians. In 2018, we published an interesting case about somatoparaphrenia with misoplegia, which was undiagnosed and neglected before admission to our neurorehabilitation unit (Maggio et al., 2020). In 2017, we published a case of a patient with spinal cord injury who was treated for gait disorders using a combination of robotic gait training and neuromodulation (rTMS) of the motor cortex (Calabrò et al., 2017). We present a summary of these cases, reporting also our diagnostic and rehabilitation process.

### 3.1 ChatGPT for clinical diagnosis and prognosis of a patient with somatoparaphrenia with misoplegia

A 50-year-old Italian woman, who worked as a teacher and translator, underwent neurosurgical intervention for a left parietal meningioma in March 2019, resulting in right hemiparesis. Despite normal cognitive function, she exhibited a reluctance to participate in lower limb exercises during rehabilitation. Brain MRI showed post-surgery changes and neuropsychological assessments revealed normal cognitive function but moderate anxiety. Spatial neglect and awareness of motor deficits were ruled out, but somatoparaphrenia (SP) and misoplegia were diagnosed, confined to the right lower limb (particularly the right foot). Treatment involved multidisciplinary approaches: psychological counseling aimed at body integration, traditional physiotherapy based on the Bobath approach, and robotic rehabilitation using Hunova technology to improve balance and muscle strength. Psychological counseling focused on body awareness and emotional experiences, while physiotherapy was aimed at reducing spasticity and improving balance. Robotic rehabilitation targeted balance, limb strengthening, and trunk control customized over 8 weeks, three times weekly. Therefore, our integrated approach (psychological counseling, physiotherapy, in addition to innovative neurorobotic approach) was aimed to provide holistic patient management and enhance self-body awareness, facilitating a comprehensive representation of the patient’s body (Maggio et al., 2020).

**BOX 1 Example prompts and outputs of GPT 3.5 in the context of a previously published clinical case**

**Prompt:** I would like to present you with this clinical case report. Could you help me to improve my medical diagnosis? (Maggio et al., 2020)

**ChatGPT 3.5:** Certainly, I’d be happy to help! Please provide the details of the clinical case report, including relevant patient history, symptoms, physical exam findings, laboratory results, imaging studies, and any other pertinent information. The more details you provide, the better I can assist you in improving the medical diagnosis.

**Prompt:** A 50-year-old, right-handed, Italian woman attended our Institute (in day hospital regimen) from June to September 2019, owing to a right hemiparesis after a neurosurgical intervention for a left parietal meningioma (occurred in March 2019). She was an English/French teacher and an unmarried translator and lived with her 80-year-old mother. Her family and personal history were negative for neurological and psychiatric disorders. At the admission in Hospital, the patient presented with difficulty standing in the upright position, abnormal gait and right moderate hemiparesis, mainly involving the lower limb. No alteration of proprioception or sense of vibration was observed. No cognitive deficits were reported after the surgical intervention and during the rehabilitation period in another Hospital. However, when reviewing her past rehab history, we found that the patient systematically refused the exercises focused on her lower limb. No one investigated the reason why she behaved this way. The patient also reported that this was the first time she talked about this problem. A brain magnetic resonance scan revealed the results of the parietal vertex craniotomy, with the surgical cavity surrounded by peripheral gliotic reaction and Wallerian degeneration of the left corticospinal tract. No gadolinium enhancement was appreciable.

**ChatGPT 3.5:** Thank you for providing the detailed clinical case report. Based on the information provided, it seems that the patient is experiencing right hemiparesis following a neurosurgical intervention for a left parietal meningioma. Here are some key points to consider:

Clinical presentation: The patient presents with difficulty standing upright, abnormal gait, and right moderate hemiparesis, particularly affecting the lower limb. No alteration of proprioception or sense of vibration is observed. The patient did not report any cognitive deficits after the surgical intervention.

Rehabilitation history: The patient has a history of systematically refusing exercises focused on her lower limb during rehabilitation in another hospital. The reason for her refusal has not been investigated previously.

Neuroimaging findings: Brain magnetic resonance scan reveals the results of the parietal vertex craniotomy. Surgical cavity surrounded by peripheral gliotic reaction and Wallerian degeneration of the left corticospinal tract. No gadolinium enhancement is appreciable.

Social and personal history: The patient is a 50-year-old, right-handed, Italian woman who was an English/French teacher and translator. She lives with her 80-year-old mother, and there is no significant family history of neurological or psychiatric disorders.

Impressions and considerations: The neurological deficits observed can be correlated with the location of the meningioma and the subsequent surgical intervention. Wallerian degeneration of the left corticospinal tract may contribute to the observed hemiparesis. The patient’s refusal of lower limb exercises during rehabilitation raises questions about potential psychological or behavioral factors that may be impacting her recovery. A multidisciplinary approach involving neurology, rehabilitation medicine, and psychology may be beneficial in addressing both the physical and psychological aspects of her recovery.

Recommendations: Consider a thorough psychological assessment to explore the reasons behind the patient’s refusal of lower limb exercises. Collaborate with a rehabilitation team to tailor an exercise program that addresses the patient’s concerns and encourages active participation. Regular follow-up neuroimaging and clinical assessments to monitor the progress of Wallerian degeneration and overall recovery. Engage the patient in open communication regarding her rehabilitation goals and expectations. It’s important to note that the above considerations are based on the provided information, and a comprehensive evaluation by the treating medical team would be essential for a more accurate diagnosis and treatment plan.

**Prompt:** Thanks a lot, we have performed a psychological evaluation revealing additional information about her relationship with the lower left limb. She feels a sense of disgusting, lack of confidence regarding her left lower foot. She thought that her left foot could have betrayed her.

**ChatGPT 3.5:** Thank you for providing additional information from the psychological evaluation. It appears that the patient’s aversion and lack of confidence in her left lower limb may be contributing to her refusal of exercises and the observed difficulties in gait and lower limb function. This psychological aspect is crucial to address for a comprehensive treatment plan. Here are some considerations:

Body image and self-perception: The patient’s feelings of disgust and lack of confidence related to her left lower foot suggest a potential body image disturbance. Explore the patient’s beliefs and perceptions about her body post-surgery and how these emotions may be influencing her rehabilitation.

Psychological support: Engage the patient in open and empathetic communication to understand the underlying fears and concerns related to her left lower limb. Consider involving a psychologist or mental health professional experienced in rehabilitation to work with the patient on addressing her emotional challenges.

Gradual exposure and desensitization: Develop a structured and gradual exposure plan to help the patient become more comfortable with and accepting of her left lower limb. Encourage small, manageable steps to build confidence and gradually reintroduce exercises focusing on the lower limb.

Collaboration with rehabilitation team: Ensure close collaboration between the rehabilitation team, including physical therapists, occupational therapists, and psychologists, to create an integrated approach to address both physical and psychological aspects.

Education and empowerment: Provide the patient with education about the recovery process, the role of rehabilitation exercises, and the potential for improvement with consistent effort. Empower the patient by involving her in the goal-setting process and highlighting achievable milestones.

Family support: Involve the patient’s family, particularly her 80-year-old mother, in the rehabilitation process to provide emotional support and encouragement.

Ongoing assessment: Conduct regular assessments to track the patient’s progress, both in terms of physical function and psychological well-being. Remember, a collaborative and patient-centered approach that addresses both the physical and psychological aspects of recovery is essential for the best possible outcomes. Each step should be taken at a pace that the patient is comfortable with, and the rehabilitation plan should be adapted based on her responses and needs.

**Prompt:** Ok, could you now give me a final diagnosis of this patient?

**ChatGPT 3.5:** I’m not a substitute for a qualified medical professional, and making a final diagnosis requires a thorough examination of the patient, including in-person assessments and consultations with relevant specialists. However, based on the information provided, I can offer a provisional diagnostic impression that may guide further evaluation and management:

Provisional diagnosis: post-surgical left parietal meningioma sequelae: The patient’s right hemiparesis and neurological deficits are likely sequelae of the left parietal meningioma and subsequent surgical intervention. Wallerian degeneration of the left corticospinal tract contributes to the observed motor impairment.

Psychological factors affecting rehabilitation: Body image disturbance, feelings of disgust, and lack of confidence regarding the left lower limb are significant psychological factors impacting rehabilitation. The patient’s fear of betrayal by her left foot may be contributing to her refusal of lower limb exercises.

Phobia or avoidant behavior related to left lower limb: The patient’s aversion and avoidance of exercises targeting the left lower limb may be indicative of a specific phobia or avoidant behavior.

Recommendations for further evaluation and management:

Neurological assessment: Continue regular neurological assessments to monitor the progression of Wallerian degeneration and identify any additional neurological changes.

Psychological assessment: Further explore the psychological aspects affecting the patient’s rehabilitation, considering a formal psychological evaluation.

Collaborative rehabilitation plan: Develop a comprehensive and collaborative rehabilitation plan involving physical therapy, occupational therapy, and psychology to address both physical and psychological aspects.

Gradual exposure and desensitization: Implement a structured plan for gradual exposure and desensitization to address the patient’s aversion to her left lower limb.

Family involvement: Involve the patient’s family in providing emotional support and encouragement throughout the rehabilitation process.

It is crucial to involve a multidisciplinary team, including neurologists, rehabilitation specialists, psychologists, and potentially psychiatrists, to ensure a holistic approach to the patient’s care. The final diagnosis and treatment plan should be determined through ongoing evaluation and collaboration with the patient and her healthcare team.

**Prompt:** Your diagnosis is too widespread. The patient would seem to not respond classical neurorehabilitation protocols. For this reason, I need a suggestion for additional clinical assessment in order to improve diagnosis and prognosis.

**ChatGPT 3.5:** Certainly, if the patient is not responding to classical neurorehabilitation protocols and there are unique psychological factors influencing her rehabilitation, further assessments may be warranted. Here are some additional clinical assessments that may provide valuable insights:

Functional MRI (fMRI): Consider functional MRI to assess brain activity during motor tasks, providing information on neural connectivity and potential compensatory mechanisms.

Neuropsychological assessment: Perform a comprehensive neuropsychological assessment to evaluate cognitive functions, emotional well-being, and the impact of psychological factors on daily functioning.

Electromyography (EMG) and nerve conduction studies: Conduct EMG and nerve conduction studies to assess the integrity and function of peripheral nerves and muscles, especially in the affected limb.

Mirror therapy: Explore the use of mirror therapy as a potential intervention. This technique uses a mirror to create the illusion of movement in the affected limb, potentially helping to alleviate discomfort and improve motor function.

Virtual reality (VR) rehabilitation: Consider incorporating virtual reality-based rehabilitation programs to engage the patient in a more interactive and immersive rehabilitation experience.

Pain assessment: Assess the presence of any pain in the affected limb, as pain can significantly impact motor function and rehabilitation outcomes.

Orthopedic evaluation: Include an orthopedic evaluation to rule out any musculoskeletal issues that might contribute to the observed difficulties in gait and lower limb function.

Psychiatric consultation: Consult with a psychiatrist to assess for any underlying psychiatric conditions, such as anxiety or depression, that may be influencing the patient’s response to rehabilitation.

Biofeedback: Implement biofeedback techniques to provide real-time information about physiological processes (e.g., muscle activity) and assist in self-regulation.

These additional assessments aim to provide a more nuanced understanding of the factors contributing to the patient’s challenges and may guide the development of a tailored and effective rehabilitation plan. Collaborating with specialists from various disciplines will enhance the holistic care approach and improve the chances of a successful outcome.

**Prompt:** Following DMS-IV-TR criteria, How could this patient be considered?

**ChatGPT 3.5:** The DSM-IV-TR is a manual published by the American Psychiatric Association and provides criteria for the classification of mental disorders. Given the information provided, it’s important to note that I’m not a mental health professional, and a definitive diagnosis requires a thorough evaluation by qualified clinicians. However, based on the information available, the patient’s psychological symptoms may be indicative of specific conditions or considerations. One possible consideration is the presence of a specific phobia or a related anxiety disorder.

Potential considerations: specific phobia: The patient’s aversion, feelings of disgust, and lack of confidence regarding her left lower limb could be consistent with specific phobia criteria. Specific phobia involves a marked and persistent fear of a specific object or situation.

Avoidant behavior: The patient’s systematic refusal of exercises targeting the left lower limb may be indicative of avoidant behavior related to the specific phobia. Avoidant behavior is a common feature in phobic disorders.

Adjustment disorder: The patient’s psychological distress following the neurosurgical intervention and her reluctance to engage in rehabilitation exercises might be indicative of an adjustment disorder. Adjustment disorders can occur in response to a significant life stressor, such as a medical illness or surgery.

Other specified anxiety disorder: Given the unique nature of the patient’s psychological distress, a broader consideration might be an “other specified anxiety disorder” if the symptoms do not precisely fit the criteria for a specific phobia.

It’s crucial to involve a mental health professional, such as a psychiatrist or clinical psychologist, for a thorough assessment based on DSM-IV-TR criteria. They can conduct interviews, administer standardized assessments, and consider the patient’s overall clinical presentation to arrive at an accurate diagnosis. Additionally, the DSM-5 has been released since the DSM-IV-TR, and clinicians now primarily use the DSM-5 for diagnostic purposes. Therefore, it’s essential to consider the latest edition for a comprehensive evaluation.

**Prompt:** For my opinion this patient could have a body image disturbance. Isn’t?

**ChatGPT 3.5:** Yes, your observation aligns with the possibility of a body image disturbance in this patient. Body image disturbance refers to a person’s subjective perception, thoughts, and feelings about their own body. In this case, the patient’s feelings of disgust, lack of confidence, and the belief that her left foot could betray her suggest a negative and distorted perception of her own body, specifically related to the left lower limb. This body image disturbance could be contributing to her avoidance of exercises focused on the left lower limb during rehabilitation. Addressing these psychological factors, such as body image concerns, is crucial for a comprehensive and effective treatment plan. Mental health professionals, including clinical psychologists or psychiatrists, can further assess and provide targeted interventions to help the patient overcome these challenges and improve her engagement in rehabilitation.

It’s important to explore the underlying reasons for her negative body image and work collaboratively with both neurological and psychological perspectives to enhance her overall well-being and rehabilitation outcomes.

**Prompt:** Following the first impression of neurorehabilitation team this patient could be a case of somatoparaphrenia with misoplegia.

**ChatGPT 3.5:** Somatoparaphrenia is a neuropsychiatric syndrome characterized by a delusional belief that one’s limb or limbs do not belong to one’s own body. Misoplegia, on the other hand, refers to a strong dislike or aversion to a paralyzed limb. Both conditions are typically associated with brain lesions, often in the right parietal lobe. Given the information provided, especially the patient’s aversion, feelings of disgust, and lack of confidence regarding her left lower limb, somatoparaphrenia and misoplegia could be considered as potential diagnostic considerations. These conditions may manifest as a result of the surgical intervention and associated brain changes. It’s crucial to involve a neurologist or a neuropsychiatrist for a detailed assessment, including neuroimaging studies and a thorough clinical examination, to confirm or rule out somatoparaphrenia and misoplegia. The interdisciplinary collaboration between neurology, rehabilitation, and psychiatry is essential for comprehensive care and developing an appropriate treatment plan that addresses both the neurological and psychological aspects of the patient’s condition.

**Prompt:** Which is the best neurorehabilitation plan for this kind of patient?

**ChatGPT 3.5:** Designing an effective neurorehabilitation plan for a patient with somatoparaphrenia and misoplegia involves addressing both the neurological deficits and the associated psychological factors. Here are some key components to consider:

Interdisciplinary team collaboration: Assemble an interdisciplinary team including neurologists, physical therapists, occupational therapists, neuropsychologists, and psychiatrists to provide comprehensive care.

Neurological rehabilitation: Focus on traditional neurological rehabilitation to improve motor function, strength, and coordination in the affected limb. Implement targeted exercises and activities to address specific motor deficits associated with somatoparaphrenia and misoplegia.

Mirror therapy: Consider incorporating mirror therapy, a technique where a mirror is used to create the illusion of movement in the affected limb. This may help alleviate the aversion and improve body perception.

Virtual reality (VR) rehabilitation: Utilize virtual reality-based rehabilitation programs to create engaging and immersive experiences, promoting motor learning and reducing psychological barriers.

Graded motor imagery (GMI): GMI is a step-by-step process involving laterality recognition, motor imagery, and mirror therapy. It can be effective in addressing body schema issues associated with somatoparaphrenia.

Cognitive behavioral therapy (CBT): Integrate CBT or other psychotherapeutic approaches to address the patient’s negative beliefs and emotions related to the affected limb. Work on challenging and modifying maladaptive thoughts and behaviors associated with somatoparaphrenia.

Exposure therapy: Gradual exposure to the affected limb and activities may help desensitize the patient to the aversive feelings and improve acceptance.

Patient education: Provide education to the patient and their family about the nature of somatoparaphrenia and misoplegia, emphasizing the collaborative and supportive role of the rehabilitation team.

Family involvement: Engage the patient’s family in the rehabilitation process to provide emotional support and encouragement, as family support can be crucial in the recovery journey.

Ongoing assessment and adjustment: Regularly assess and adjust the rehabilitation plan based on the patient’s progress and responses.

Encourage open communication to address any concerns or challenges the patient may be facing. The neurorehabilitation plan should be tailored to the individual needs of the patient, taking into account the specific manifestations of somatoparaphrenia and misoplegia. Regular collaboration and communication between team members will enhance the overall effectiveness of the rehabilitation process. Additionally, the plan should be dynamic, allowing for adjustments based on the patient's evolving needs and progress.

**Prompt:** Following your suggestion, this patient has been assigned to a novel multidisciplinary treatment, including motor rehabilitation training, traditional physiotherapy and robotic rehabilitation using the Hunova Movendo Technology and psychological counseling. We observed a significant clinical improvement in somatoparaphrenia symptoms. What you think about that? Could we include additional treatments?

**ChatGPT 3.5:** It’s encouraging to hear that the patient has shown significant clinical improvement in somatoparaphrenia symptoms with the novel multidisciplinary treatment approach. The combination of motor rehabilitation training, traditional physiotherapy, robotic rehabilitation using Hunova Movendo Technology, and psychological counseling is comprehensive and addresses both the neurological and psychological aspects of the condition. Considering the positive response, there is always room for ongoing evaluation and potential adjustments to optimize the treatment plan further. Here are some considerations and potential additional treatments:

Continued psychological support: Continue and potentially intensify psychological counseling to address any residual psychological factors contributing to somatoparaphrenia. Implement strategies for coping with stress and anxiety, as emotional well-being is crucial in the overall recovery process.

Biofeedback and neurofeedback: Consider incorporating biofeedback or neurofeedback techniques to provide real-time information about physiological processes (e.g., muscle activity, brainwaves). These techniques may enhance the patient’s awareness and control over physiological responses, potentially aiding in motor rehabilitation.

Mindfulness-based interventions: Introduce mindfulness-based interventions, such as mindfulness meditation or mindful movement exercises, to enhance body awareness and promote a positive connection with the affected limb.

Group therapy or support groups: Explore the possibility of group therapy or support groups where the patient can share experiences and strategies with others facing similar challenges. Peer support can be valuable in reinforcing positive changes and promoting a sense of community.

Cognitive remediation therapy: If there are cognitive deficits or challenges related to body schema perception, consider cognitive remediation therapy to address cognitive processes and improve functional outcomes.

Community integration activities: Plan activities that involve community integration to enhance the patient’s confidence and social participation. This could include supervised outings or participation in community events.

Regular assessments and feedback: Continue regular assessments to monitor progress and gather feedback from the patient about their experiences and perceived improvements. Use this information to adapt and refine the treatment plan as needed.

It’s important to maintain open communication with the patient and regularly reassess their goals and preferences. The success of the treatment plan may depend on the patient’s engagement, and tailoring interventions to their individual needs can further optimize outcomes. Additionally, ongoing collaboration among the multidisciplinary team will contribute to a holistic and effective approach to the patient’s care.

### 3.2 ChatGPT evaluating the rehabilitation planning of a patient with spinal cord injury

In 2018, we published a case report relating to a 31-year-old man affected for around 20 months by incomplete post-traumatic spinal cord injury (Calabrò et al., 2017). After an initial neurorobotic treatment with the Lokomat system, this patient did not show significant clinical improvement in gait recovery. For this reason, we decided to refine our previous rehabilitation plan with a different approach combining neurorobotic device with repetitive transcranical magnetic stimulation (rTMS). In a second session, we implemented an identical Lokomat session paired to an rTMS paradigm over the primary motor cortex, to ameliorate patient’s outcomes. After this second combined treatment, we observed a significant amelioration of gait scales, and a statistically significant reduction of hip and knee stiffness, device guidance force, BWS and an increase of hip flexion-extension force, MEP amplitude, and speed.

**BOX 2 Example prompts and outputs of GPT 3.5 in the context of a previously published clinical case**

**Prompt:** I would like to present you with this clinical case report. Could you help me to improve my rehabilitation plan? (Calabrò et al., 2017)

**ChatGPT 3.5:** Of course! I’d be happy to help you with your clinical case report and offer suggestions to improve your rehabilitation plan. Please provide the details of the case report along with the current rehabilitation plan, and let me know specifically what aspects you are looking to improve or refine.

**Prompt:** A 31-year-old man affected for around 20 months by incomplete post-traumatic spinal cord injury following a motorbike accident, came to our observation for an intensive neurorehabilitation treatment. The neurological examination showed a moderate to severe paraparesis, hypoesthesia below the umbilicus, urge incontinence, and erectile dysfunction (American Spinal Injury Association Impairment Scale-ASIA = C; Lower Extremity Motor Score-LEMS = 3). A spinal cord MRI-scan showed a T2-hyperintense lesion at T10. The lower-limb somatosensory evoked potentials showed a P40 latency of 46 (right) and 45 ms (left) (N22 of 21.4 and 21.5 ms, respectively) and bilateral amplitude of ∼4 μV, whereas the motor central conduction time (CCT) was bilaterally ∼37 ms. Which form of neurorehabilitation therapy would you recommend?

**ChatGPT 3.5:** Given the clinical presentation of incomplete post-traumatic spinal cord injury with moderate to severe paraparesis, hypoesthesia, urinary incontinence, erectile dysfunction, and ASIA grade C classification, a comprehensive neurorehabilitation program is crucial to optimize functional outcomes and quality of life for the patient. Here are some recommendations for the neurorehabilitation therapy:

1. Physical therapy: Focus on improving strength, flexibility, and endurance of the lower extremities through targeted exercises and activities. Incorporate functional tasks such as sit-to-stand transfers, walking with assistive devices, and balance training to promote independence in mobility.
2. Occupational therapy: Address activities of daily living (ADLs) and upper extremity function to enhance the patient’s ability to perform self-care tasks independently. Adaptive equipment and techniques may be introduced to compensate for functional limitations.
3. Functional electrical stimulation (FES): Utilize FES to facilitate muscle activation and improve motor function in the lower limbs. FES can be applied during gait training to assist with muscle contraction and promote a more natural walking pattern.
4. Aquatic therapy: Take advantage of the buoyancy and resistance provided by water to facilitate movement and reduce the risk of injury during exercise. Aquatic therapy can improve strength, endurance, and flexibility while minimizing stress on weight-bearing joints.
5. Bladder and bowel management program: Implement a structured program to manage urinary incontinence and bowel dysfunction, which may include timed voiding, pelvic floor exercises, and bowel training techniques.
6. Sexual rehabilitation: Address erectile dysfunction through counseling, education, and potentially, pharmacological interventions. Collaborate with specialists in sexual medicine to develop a tailored treatment plan.
7. Psychological support: Offer psychological counseling and support to address emotional and psychological challenges associated with spinal cord injury, such as depression, anxiety, and adjustment issues. Encourage participation in peer support groups and community resources.
8. Gait training with body weight support (BWS): Incorporate gait training with BWS using specialized equipment such as a treadmill with overhead harness support. This approach allows for repetitive practice of walking tasks while providing adjustable levels of weight support to promote safe and effective gait training.
9. Neurostimulation techniques: Consider transcutaneous electrical nerve stimulation (TENS) or neuromuscular electrical stimulation (NMES) to modulate pain perception, improve circulation, and enhance motor recovery.
10. Robotic-assisted rehabilitation: Integrate robotic devices into the rehabilitation program to facilitate repetitive movement training, enhance muscle strength, and promote motor learning. Robotic exoskeletons or orthoses can assist with gait retraining and support the patient during upright mobility tasks.
11. Continuity of care and long-term follow-up: Establish a comprehensive discharge plan and provide resources for ongoing community-based rehabilitation services, home modifications, and adaptive equipment as needed. Schedule regular follow-up appointments to monitor progress, address evolving needs, and optimize outcomes over time.

It’s important to individualize the rehabilitation plan based on the patient’s specific goals, functional abilities, and preferences, while also considering interdisciplinary collaboration and holistic care approaches. Regular reassessment and adjustment of the treatment plan are essential to maximize the patient’s potential for recovery and adaptation to the consequences of spinal cord injury.

**Prompt:** Thanks a lot. we chose to perform a robotic-assisted rehabilitation program with Lokomat using the Augmented Performance Feedback (APF) modality (i.e., the LokomatPro) that provides motivating, challenging and instructive functional feedback in virtual environments. The patient performed a 40 min session per day, between 9 A.M. and 12 P.M., from Monday to Friday, for 8 consecutive weeks, for a total of 40 sessions. The amount of BWS was initially set at 70% of the patient’s weight, then decreased according to the patient’s load tolerance (61 ± 6%), and the gait speed was adjusted in order to make the exercise comfortable for the patient (1.5 ± 0.3 km/h). After the first neurorobotic session, the patient showed a very mild improvement concerning kinetic parameters (reduction of hip and knee stiffness), and non-significant clinical and electrophysiological changes. For this reason, for a follow-up rehabilitation session, could I inquire about additional or different forms of treatment that you would recommend?

**ChatGPT 3.5:** Given the initial response to the robotic-assisted rehabilitation program with Lokomat using augmented performance feedback (APF) modality, it’s important to reassess the treatment plan and consider additional or alternative interventions to optimize the patient’s progress. Here are some suggestions for further rehabilitation strategies:

1. Combination therapy: Incorporate a multimodal approach by combining Lokomat training with other therapeutic modalities such as functional electrical stimulation (FES), manual therapy, or gait training with body weight support (BWS) on a treadmill. This can provide a more comprehensive and synergistic effect on motor recovery.
2. Task-specific training: Emphasize task-specific activities and functional movements that are meaningful to the patient’s daily life. Focus on activities such as sit-to-stand transfers, stair negotiation, and overground walking to improve functional mobility and independence.
3. Virtual reality rehabilitation: Explore the use of virtual reality (VR) technology to enhance engagement, motivation, and neuroplasticity during rehabilitation sessions. VR-based interventions can provide immersive environments and interactive tasks to simulate real-life scenarios and promote motor learning.

**ChatGPT 3.5:**

Manual therapy and stretching: Incorporate manual therapy techniques such as joint mobilizations, soft tissue mobilization, and stretching to address muscle tightness, improve range of motion, and optimize biomechanical alignment.

1. Functional neuromuscular stimulation (FNS): Consider integrating FNS to activate paralyzed muscles and facilitate voluntary motor control. FNS can be used in conjunction with Lokomat training to enhance muscle recruitment patterns and promote functional recovery.
2. Biofeedback training: Utilize biofeedback techniques to provide real-time visual or auditory feedback on muscle activation and movement patterns. Biofeedback training can help the patient to improve motor control, coordination, and muscle recruitment strategies.
3. Home exercise program: Develop a customized home exercise program that complements the in-clinic rehabilitation sessions and encourages active participation in self-management of the condition. Provide clear instructions and supervision to ensure safety and adherence to the program.
4. Psychosocial support and counseling: Continue to offer psychological support and counseling to address any emotional, social, or psychological challenges that may impact the patient’s rehabilitation journey. Encourage open communication and collaboration with the interdisciplinary team.
5. Nutritional counseling: Consider consulting with a dietitian or nutritionist to optimize the patient’s nutritional status and support overall health and well-being. Proper nutrition plays a crucial role in promoting recovery and enhancing physical function.
6. Reassessment and goal setting: Conduct regular reassessment of the patient’s functional status, goals, and progress to guide the modification of the rehabilitation plan as needed. Set realistic and achievable goals in collaboration with the patient to maintain motivation and momentum throughout the rehabilitation process.

By integrating these additional or alternative forms of treatment into the rehabilitation plan, you can provide a more comprehensive and individualized approach to address the patient’s specific needs and optimize outcomes. Regular monitoring and adjustment of the treatment plan based on the patient’s response and progress are essential to maximize the effectiveness of rehabilitation interventions.

**Prompt:** Thanks a lot for your suggestions. However, since the use of the Lokomat device alone—which consists of powered gait orthoses with integrated computer-controlled linear actuators at each hip and knee joint, a BWSS, and a treadmill—is insufficient to induce a consistent gait recovery, I would like to know if you could recommend any additional or alternative forms of gait treatment.

**ChatGPT 3.5:** Given the initial response to the robotic-assisted rehabilitation program with Lokomat using augmented performance feedback (APF) modality, it’s important to reassess the treatment plan and consider additional or alternative interventions to optimize the patient’s progress. Here are some suggestions for further rehabilitation strategies:

1. Combination therapy: Incorporate a multimodal approach by combining Lokomat training with other therapeutic modalities such as functional electrical stimulation (FES), manual therapy, or gait training with body weight support (BWS) on a treadmill. This can provide a more comprehensive and synergistic effect on motor recovery.
2. Task-specific training: Emphasize task-specific activities and functional movements that are meaningful to the patient’s daily life. Focus on activities such as sit-to-stand transfers, stair negotiation, and overground walking to improve functional mobility and independence.
3. Virtual reality rehabilitation: Explore the use of virtual reality (VR) technology to enhance engagement, motivation, and neuroplasticity during rehabilitation sessions. VR-based interventions can provide immersive environments and interactive tasks to simulate real-life scenarios and promote motor learning.
4. Manual therapy and stretching: Incorporate manual therapy techniques such as joint mobilizations, soft tissue mobilization, and stretching to address muscle tightness, improve range of motion, and optimize biomechanical alignment.
5. Functional neuromuscular stimulation (FNS): Consider integrating FNS to activate paralyzed muscles and facilitate voluntary motor control. FNS can be used in conjunction with Lokomat training to enhance muscle recruitment patterns and promote functional recovery.
6. Biofeedback training: Utilize biofeedback techniques to provide real-time visual or auditory feedback on muscle activation and movement patterns. Biofeedback training can help the patient to improve motor control, coordination, and muscle recruitment strategies.
7. Home exercise program: Develop a customized home exercise program that complements the in-clinic rehabilitation sessions and encourages active participation in self-management of the condition. Provide clear instructions and supervision to ensure safety and adherence to the program.
8. Psychosocial support and counseling: Continue to offer psychological support and counseling to address any emotional, social, or psychological challenges that may impact the patient’s rehabilitation journey. Encourage open communication and collaboration with the interdisciplinary team.
9. Nutritional counseling: Consider consulting with a dietitian or nutritionist to optimize the patient’s nutritional status and support overall health and well-being. Proper nutrition plays a crucial role in promoting recovery and enhancing physical function.
10. Reassessment and goal setting: Conduct regular reassessment of the patient’s functional status, goals, and progress to guide the modification of the rehabilitation plan as needed. Set realistic and achievable goals in collaboration with the patient to maintain motivation and momentum throughout the rehabilitation process.

By integrating these additional or alternative forms of treatment into the rehabilitation plan, you can provide a more comprehensive and individualized approach to address the patient’s specific needs and optimize outcomes. Regular monitoring and adjustment of the treatment plan based on the patient’s response and progress are essential to maximize the effectiveness of rehabilitation interventions.

### 3.3 Considerations for clinical scenarios

In the first case, we compared the diagnostic ability of GPT-3.5 and assessed its ability to suggest possible treatments. In the second case, we compared a senior healthcare physician’s ability to define new combinations of different neurorehabilitation treatments with that of GPT-3.5. We observed that with a clear query, GPT-3.5 approached the diagnostic evaluation by defining the possibility of anxiety or body representation disorder. However, unlike a clinician, GPT-3.5 did not provide a clear diagnosis and overlooked aspects that can only be defined through a psychological interview and objective examination Similarly, in the second case, GPT-3.5 did not consider the possibility to combine the neurorobotic treatment with rTMS. While GPT-3.5 sufficiently directs a clinician in the adequate diagnostic and rehabilitation pathways, it is evident that that it cannot replace a real therapist/physician, although we were surprised to observe that ChatGPT provided indications of an interdisciplinary and multimodal approach, which encompassed psychological aspects, traditional physical therapy, and innovative methods. Indeed, in the rehabilitation of this case, our team collaborated in an integrated way, including the neurologist, physiatrist, psychologist, and physiotherapist with expertise in both conventional and robotic approaches. However, it should be noted that in the first case, we promoted an integrated approach with robotics, instead of virtual reality as suggested by GPT-3.5, to encourage strengthening and confidence in the patient’s right foot/ankle, combining psychotherapeutic activities.

Overall, we found that ChatGPT performed a good approximation for diagnosis and rehabilitation in the complex challenges posed by the cases. However, it is important to note that ChatGPT did not perform as well as our management, particularly in tailoring rehabilitation practices to the patient’s needs (see Box 2). While GPT-3.5 is not specifically designed for medical tasks, ChatGPT may hold promise for guiding appropriate clinical stream but may not be suitable for making decisions in those contexts. Further studies are certainly needed, including comparisons of multiple cases, with adequate statistical analysis that includes accuracy, reliability, and other statistical indices to ascertain the true feasibility of using this tool in clinical practice.

Finally, although our study used GPT-3.5, updated through 2021, it is critical to realize that medical technologies are continually advancing. As recently demonstrated (Lu et al., 2023; Cerasa and Crowe, 2024; Eriksen et al., 2024), ChatGPT’s newer versions—like GPT-4—performed better than its predecessors. Consequently, once the most recent NLP developments are more widely available, research in the future should take them into account.

## 4 Future directions

As demonstrated in the previous example, ChatGPT has the potential to be considered a facilitator of decision-making in clinical practice. However, it’s crucial to keep in mind that for generative AI to properly work, domain-specific knowledge is necessary. To date, there has been no empirical data demonstrating ChatGPT’s effectiveness in answering medical queries related to neurorehabilitation. Therefore, similar to what is being done in other medical fields (i.e., oncology, neurology), the performance of ChatGPT in the diagnosis and prognosis of ABI patients needed to be benchmarked (Vat et al., 2021; Clusmann et al., 2023; Dahmen et al., 2023; De Angelis et al., 2023). Following the methodological approach of Huang et al. (2023) we need to evaluate the validity and precision in medical diagnosis and therapy using diverse, high-quality, shared, and worldwide recognized standardized clinical tests of expert-level knowledge. For example, in the study by Huang et al. (2023), ChatGPT 4.0 was benchmarked against ChatGPT 3.5 using the 38th American College of Radiology radiation oncology in-training exam. The results showed that ChatGPT 4.0 achieved higher accuracies (74.6%) compared to ChatGPT 3.5 (63.65%), demonstrating its effectiveness in diagnosing and prognosing oncological conditions.

Neurorehabilitation is a complex medical field aimed at aiding recovery from nervous system injury and minimizing and/or compensating for any functional alterations resulting from it. With the advent of ChatGPT, several steps underlying the decision-making processes could be supported. Indeed, ChatGPT’s capabilities can be harnessed in neurorehabilitation to assist with tasks such as planning agendas, booking appointments, and managing medication schedules for individuals recovering from brain injuries or strokes (Peng et al., 2024). Its linguistic abilities can facilitate conversation, which is particularly beneficial for patients with speech or language deficits. The AI can suggest exercises tailored to each patient’s unique needs, considering factors like age, socioeconomic status, and specific physical, mental, and neurological conditions (Bandi et al., 2023). ChatGPT can also provide explanations on how and why certain exercises should be performed, potentially enhancing patient understanding and compliance. In speech therapy, ChatGPT can be used to generate therapy materials and provide practice exercises outside of therapy sessions. It can also assist speech-language pathologists (SLPs) by reducing workload and therapy preparation costs.

## 5 ChatGPT: ethical consideration and limitations

The use of ChatGPT in the medical field raises important ethical and practical questions (Floridi et al., 2018). First, ChatGPT may lack the ability to suggest humanistic, non-pharmacological approaches (Roy et al., 2024; Suri et al., 2024). This is attributed to its learning method, mainly based on pattern recognition and statistical associations (Chen et al., 2024), as discussed in a study conducted by Suri et al. (2024). Consequently, the responses generated may be too structured and lack human sensitivity.

Furthermore, research has raised concerns regarding the accuracy of the clinical information covered by ChatGPT, as erroneous data could lead to misinterpretations (Mittal and Dhar, 2023). This raises further ethical concerns regarding the security and integrity of medical information processed by AI, as noted by Roman et al. (2023). Therefore, the use of ChatGPT as a decision-making tool requires special caution and verification by qualified medical professionals (Roy et al., 2024; Suri et al., 2024). Indeed, to ensure patient safety and the effectiveness of therapy, it is imperative to ensure that ChatGPT is used in conjunction with expert supervision (Rodriguez et al., 2024). Despite its potential to suggest multidisciplinary management strategies for medical problems, ChatGPT may still lack detailed information on potential drug side effects, making input from medical specialists indispensable (Brown et al., 2023; Cheng et al., 2023b). Thus, ChatGPT can be useful as an initial screening tool, but it is essential to recognize and manage its limitations, keeping human involvement and adequate medical supervision at the center of the decision-making process.

Finally, it is crucial to make sure that the ethics of care and the concept of patient autonomy are upheld, as well as to thoroughly analyze the implications of giving an automated system control over medical decisions. The definition of established ethical review standards for AI bots such as ChatGPT is mandatory, even though several initiatives and organizations are working to develop guidelines for ethical concerns in using AI-based algorithms in medicine (Floridi et al., 2018). Wang et al. (2023) recently conducted an in-depth examination of the ethical implications of ChatGPT in the healthcare industry, including (a) determining legal responsibility in cases where ChatGPT advice results in harm or adverse outcomes; (b) the collection and storage of sensitive patient information, which raises privacy issues, and (c) the necessity of taking health care professionals’ licensing and regulatory requirements into account when integrating ChatGPT into clinical practice.

## 6 Conclusion

In conclusion, following evidence provided by other clinical domains and the preliminary studies described in this scoping review, ChatGPT has the potential to transform rehabilitation therapy by providing both physicians and patients with individualized, engaging, and easily accessible support. Healthcare providers can assist in bridging the gap between the demand for rehabilitation services and the supply of licensed therapists by using these AI-driven solutions in the clinical stream of neurorehabilitation. More cutting-edge uses of AI in rehabilitation therapy are probably in store as the technology develops, which will ultimately improve patient outcomes and change the way we think about healthcare.

Despite its role in medical education to maintain students, physicians, nurses, and other healthcare professionals informed about new advancements and updates in their professions (Dave et al., 2023), one additional advantage of this tool will be its flexibility in meeting the specific requirements of each patient receiving rehabilitation therapy. By gaining knowledge from the patient’s advancement and inclinations, the AI model could be able to offer tailored advice and assistance. This degree of personalization has the potential to increase patients’ motivation and sense of engagement, which would ultimately improve outcome. Additionally, ChatGPT could reduce the work rehabilitation therapists have to do by automating some of the most labor-intensive duties, such as keeping track of patients’ progress and sending out workout reminders. This raises the bar for rehabilitation services overall by enabling therapists to concentrate on more intricate facets of patient care.

However, the main revolution introduced by this new kind of AI-derived technology will be the employment of 24/7 access to medical information, setting appointments, and gathering patient data. In agreement with Clusmann et al. (2023) AI-related algorithms will allow us *to democratize access* to scientific evidence and the best healthcare, equal access to the health system, where the best medical care will be available for everyone. Consequently, due to minor worries or uncertainties, ChatGPT will also help lower the need for medical aid, which will contribute to alleviating hospital crowding (Sezgin, 2023).

## Author contributions

MM: Writing – original draft, Methodology. GT: Writing – review & editing. DC: Writing – original draft, Investigation. MB: Writing – original draft, Conceptualization. RB: Writing – review & editing, Data curation. LP: Writing – review & editing, Funding acquisition. GP: Writing – review & editing, Supervision. RCS: Writing – review & editing, Writing – original draft. AC: Writing – review & editing, Writing – original draft, Conceptualization.
